# New Rare Sinapoyl Acylated Flavonoid Glycosides Obtained from the Seeds of *Lepidium apetalum* Willd

**DOI:** 10.3390/molecules200813982

**Published:** 2015-08-03

**Authors:** Lifeng Han, Pingping Shi, Yongzhe Dong, Tingting Wang, Xiaoxia Li, Jia Hao, Yi Zhang, Tao Wang

**Affiliations:** 1 Tianjin Key Laboratory of TCM Chemistry and Analysis, Institute of Traditional Chinese Medicine, Tianjin University of Traditional Chinese Medicine, 312 Anshan Road, Nankai District, Tianjin 300193, China; E-Mails: hanlifeng_1@sohu.com (L.H.); 18202682964@163.com (T.W.); 2 Tianjin State Key Laboratory of Modern Chinese Medicine, Tianjin University of Traditional Chinese Medicine, 312 Anshanxi Road, Nankai District, Tianjin 300193, China; E-Mails: shipingpingtcm@163.com (P.S.); dongyongzhe44@hotmail.com (Y.D.); huifeidedouzi@yeah.net (X.L.); haojiatjcm@126.com (J.H.)

**Keywords:** *Lepidium apetalum*, seeds, sinapoyl acylated flavonoid glycosides

## Abstract

Seven new rare sinapoyl acylated flavonoid glycosides, apetalumosides A_1_ (**1**), B_8_ (**2**), B_9_ (**3**), B_10_ (**4**), B_11_ (**5**), B_12_ (**6**), and C_1_ (**7**) were isolated from the seeds of *Lepidium apetalum* Willd. Their structures were elucidated by chemical and spectroscopic methods.

## 1. Introduction

In the course of our characterization studies on bioactive constituents from *Lepidium apetalum* Willd [1], we have reported the isolation and structure elucidation of nine new flavonoid glycosides, apetalumosides A, B_1_–B_7_, and C, together with one known isolate, quercetin 3-*O*-(2,6-di-*O*-β-d-glucopyranosyl)-β-d-glucopyranoside obtained from the seeds of it. As a continuing study on *L. apetalum* seeds, we have isolated seven new rare sinapoyl acylated flavonoid glycosides, named as apetalumosides A_1_ (**1**), B_8_ (**2**), B_9_ (**3**), B_10_ (**4**), B_11_ (**5**), B_12_ (**6**), and C_1_ (**7**) from the herbal medicine. In this paper, we describe the isolation and structure elucidation of these new ones.

## 2. Results and Discussion

**Figure 1:**
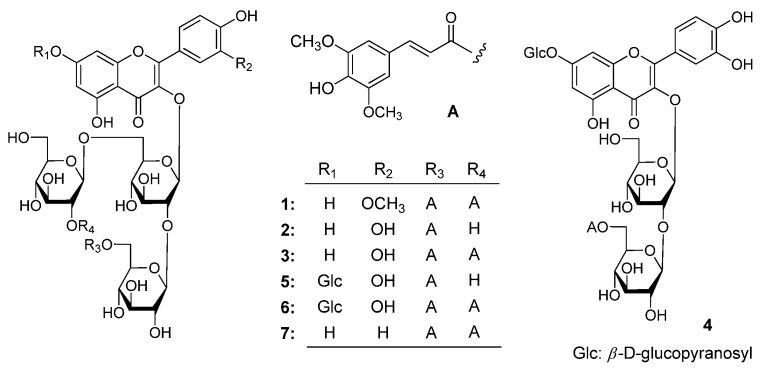
The structure of compounds **1**–**7**. (**A**), the moiety structure which indicated in the table for compounds **1**–**3** and **5**–**7**; (**4**), an isolated structure of B_10_ (**4**).

The seeds of *L. apetalum* were refluxed with 50% ethanol/water. Evaporation of the solvent under reduced pressure provided a 50% ethanol/water extract. The extract was subjected to kinds of column chromatography (CC) and finally preparative HPLC (PHPLC) to yield seven new rare sinapoyl acylated flavonoid glycosides, apetalumosides A_1_ (**1**), B_8_ (**2**), B_9_ (**3**), B_10_ (**4**), B_11_ (**5**), B_12_ (**6**), and C_1_ (**7**) (Figure 1).

**Figure 2:**
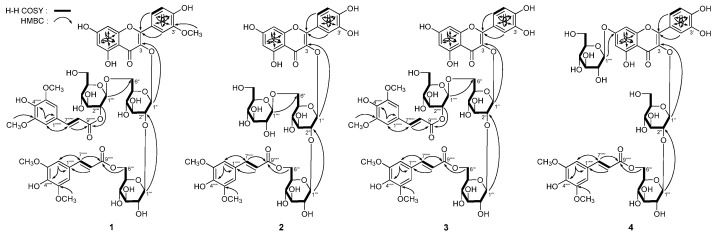
The main ^1^H ^1^H COSY and HMBC correlations of **1**–**4**.

*Apetalumoside A_1_* (**1**), ${\left[\text{α}\right]}_{\text{D}}^{25}$ –49.0° (MeOH), was isolated as yellow powder. The IR spectrum of **1** showed absorption bands ascribable to hydroxyl (3394 cm^−1^), α,β-unsaturated ester (1701, 1630 cm^−1^), aromatic ring (1648, 1604, 1514, 1458 cm^−1^), and *O*-glycosidic linkage (1075 cm^−1^). The molecular formula, C_56_H_62_O_30_, of **1** was eatablished by negative-ion HRESI-TOF-MS (*m*/*z* 1213.3238 [M − H]^−^, calcd for C_56_H_61_O_30_ 1213.3253). Acid hydrolysis of it yielded D-glucose, which was identified by retention time and optical rotation using chiral detection by HPLC analysis [1,2]. The ^1^H and ^13^C-NMR (DMSO-*d*_6_, Table 1) spectra of **1** showed signals assignable to an isorhamnetin aglycon [δ 3.85 (3H, s, 3′-OCH_3_), 6.18 (1H, br. s, H-6), 6.32 (1H, br. s, H-8), 6.90 (1H, d, *J* = 8.5 Hz, H-5′), 7.60 (1H, dd, *J* = 1.5, 8.5 Hz, H-6′), 7.74 (1H, d, *J* = 1.5 Hz, H-2′), 12.74 (1H, br. s, 5-OH)], three β-d-glucopyranosyl groups [δ 4.33 (1H, d, *J* = 8.0 Hz, H-1′′′′), 4.67 (1H, d, *J* = 7.5 Hz, H-1′′′), 5.74 (1H, d, *J* = 7.0 Hz, H-1′′)], together with two sinapoyl {6′′′-sinapoyl: δ_H_ 3.77 (6H, s, 3′′′′′,5′′′′′-OCH_3_), 6.27 (1H, d, *J* = 15.5 Hz, H-8′′′′′), 6.81 (2H, s, H-2′′′′′,6′′′′′), 7.38 (1H, d, *J* = 15.5 Hz, H-7′′′′′), and δ_C_ 166.4 (C-9′′′′′); 2′′′′-sinapoyl: δ_H_ 3.91 (6H, s, 3′′′′′′,5′′′′′′-OCH_3_), 6.28 (1H, d, *J* = 16.0 Hz, H-8′′′′′′), 7.08 (2H, s, H-2′′′′′′,6′′′′′′), 7.51 (1H, d, *J* = 16.0 Hz, H-7′′′′′′), and δ_C_ 165.6 (C-9′′′′′′)]. The ^1^H ^1^H COSY experiment (Figure 2) on **1** indicated the presence of partial structure written in bold lines. To assign the badly overlapped protons in sugar chemical shift range, HSQC-TOCSY experiment was determined. In the HSQC-TOCSY spectrum, the correlations between the following proton and carbon pairs were observed: δ_C_ 98.2 (C-1′′) and δ_H_ 3.04 (H-4′′), 3.21 (H-5′′), 3.46 (H-3′′), 3.51 (H-2′′), 5.74 (H-1′′); δ_C_ 66.7 (C-6′′) and δ_H_ 3.04 (H-4′′), 3.21 (H-5′′), 3.50, 3.75 (H_2_-6′′); δ_C_ 103.8 (C-1′′′) and δ_H_ 3.15 (H-2′′′), 3.24 (H-4′′′), 3.25 (H-3′′′), 3.40 (H-5′′′), 4.67 (H-1′′′); δ_C_ 63.3 (C-6′′′) and δ_H_ 3.24 (H-4′′′), 3.40 (H-5′′′), 4.20, 4.27 (H_2_-6′′′); δ_C_ 100.2 (C-1′′′′) and δ_H_ 2.68 (H-5′′′′), 2.87 (H-3′′′′), 3.15 (H-4′′′′), 4.33 (H-1′′′′), 4.45 (H-2′′′′); δ_C_ 60.3 (C-6′′′′) and δ_H_ 2.68 (H-5′′′′), 2.87 (H-3′′′′), 3.15 (H-4′′′′), 3.41, 3.48 (H_2_-6′′′′). Finally, in the HMBC experiment (Figure 2), long-range correlations were observed between δ_H_ 5.74 (H-1′′) and δ_C_ 132.7 (C-3); δ_H_ 4.67 (H-1′′′) and δ_C_ 82.1 (C-2′′); δ_H_ 4.33 (H-1′′′′) and δ_C_ 66.7 (C-6′′); δ_H_ 4.20, 4.27 (H_2_-6′′′) and δ_C_ 166.4 (C-9′′′′′); δ_H_ 4.45 (H-2′′′′) and δ_C_ 165.6 (C-9′′′′′′), then the connectivities between oligoglycoside moieties and aglycon or sinapoyl groups were characterized. On the basis of above mentioned evidence, the structure of apetalumoside A_1_ (**1**) was elucidated to be isorhamnetin 3-*O*-[β-d-(2-*O*-sinapoyl)-glucopyranosyl(1→6)]-β-d-(6-*O*-sinapoyl)-glucopyranosyl(1→2)-β-d-glucopyranoside.

**Table 1:** ^1^H and ^13^C-NMR data for **1** in DMSO-*d*_6_ (500 MHz for ^1^H and 125 MHz for ^13^C).

No.	δ_C_	δ_H_ (*J* in Hz)	No.	δ_C_	δ_H_ (*J* in Hz)
2	155.9	-	4′′′	69.6	3.24 (dd, 9.0, 9.0)
3	132.7	-	5′′′	73.9	3.40 (m)
4	177.3	-	6′′′	63.3	4.20 (br. d, *ca.* 12)
5	161.1	-			4.27 (dd, 5.5, 12.0)
6	98.9	6.18 (br. s)	1′′′′	100.2	4.33 (d, 8.0)
7	164.7	-	2′′′′	73.6	4.45 (dd, 8.0, 9.0)
8	94.0	6.32 (br. s)	3′′′′	74.2	2.87 (dd, 9.0, 9.0)
9	156.2	-	4′′′′	69.9	3.15 (dd, 8.0, 9.0)
10	103.8	-	5′′′′	76.4	2.68 (m)
1′	120.9	-	6′′′′	60.3	3.41 (dd, 5.0, 11.0)
2′	112.7	7.74 (d, 1.5)			3.48 (br. d, *ca.* 11)
3′	147.0	-	1′′′′′	124.2	-
4′	149.6	-	2′′′′′,6′′′′′	106.0	6.81 (s)
5′	115.3	6.90 (d, 8.5)	3′′′′′,5′′′′′	147.9	-
6′	122.8	7.60 (dd, 1.5, 8.5)	4′′′′′	138.2	-
5-OH	-	12.74 (br. s)	7′′′′′	145.2	7.38 (d, 15.5)
3′-OCH_3_	55.7	3.85 (s)	8′′′′′	114.3	6.27 (d, 15.5)
1′′	98.2	5.74 (d, 7.0)	9′′′′′	166.4	-
2′′	82.1	3.51 (dd, 7.0, 8.5)	3′′′′′,5′′′′′-OCH_3_	55.9	3.77 (s)
3′′	76.3	3.46 (dd, 8.5, 8.5)	1′′′′′′	124.6	-
4′′	69.4	3.04 (dd, 8.5, 9.0)	2′′′′′′,6′′′′′′	106.0	7.08 (s)
5′′	78.1	3.21 (m)	3′′′′′′,5′′′′′′	148.1	-
6′′	66.7	3.50 (dd, 4.5, 11.5)	4′′′′′′	138.3	-
		3.75 (br. d, *ca.* 12)	7′′′′′′	145.3	7.51 (d, 16.0)
1′′′	103.8	4.67 (d, 7.5)	8′′′′′′	114.8	6.28 (d, 16.0)
2′′′	74.3	3.15 (dd, 7.5, 8.0)	9′′′′′′	165.6	-
3′′′	76.3	3.25 (dd, 8.0, 9.0)	3′′′′′′,5′′′′′′-OCH_3_	56.2	3.91 (s)

*Apetalumoside B_8_* (**2**), was obtained as yellow powder with negative rotation (${\left[\text{α}\right]}_{\text{D}}^{25}$ –41.6°, in MeOH). The molecular formula, C_44_H_50_O_26_, of **2** was determined by negative-ion HRESI-TOF-MS (*m*/*z* 993.2522 [M − H]^−^, calcd for C_44_H_49_O_26_ 993.2518). Acid hydrolysis of it yielded d-glucose, which was identified by the same method as **1** [1,2]. The ^1^H and ^13^C (DMSO-*d*_6_, Table 2) and various 2D NMR experiments including ^1^H ^1^H COSY, HSQC, HMBC, and HSQC-TOCSY spectra of **2** indicated the presences of a quercetin aglycon [δ 6.16 (1H, d, *J* = 2.0 Hz, H-6), 6.26 (1H, d, *J* = 2.0 Hz, H-8), 6.89 (1H, d, *J* = 8.5 Hz, H-5′), 7.55 (1H, d, *J* = 2.0 Hz, H-2′), 7.59 (1H, dd, *J* = 2.0, 8.5 Hz, H-6′), 12.64 (1H, br. s, 5-OH)], three β-d-glucopyranosyls [δ 4.03 (1H, d, *J* = 7.5 Hz, H-1′′′′), 4.69 (1H, d, *J* = 7.5 Hz, H-1′′′), 5.65 (1H, d, *J* = 7.0 Hz, H-1′′)], and a sinapoyl [δ_H_ 3.77 (6H, s, 3′′′′′,5′′′′′-OCH_3_), 6.30 (1H, d, *J* = 16.0 Hz, H-8′′′′′), 6.83 (2H, s, H-2′′′′′,6′′′′′), 7.39 (1H, d, *J* = 16.0 Hz, H-7′′′′′); δ_C_ 166.4 (C-9′′′′′)]. According to the correlations from ^1^H ^1^H COSY, HSQC, and HSQC-TOCSY experiments, the ^1^H and ^13^C-NMR data for three β-D-glucopyranosyl groups were assigned in detail. Furthermore, in the HMBC experiments, long-range correlations between δ_H_ 5.65 (H-1′′) and δ_C_ 132.8 (C-3); δ_H_ 4.69 (H-1′′′) and δ_C_ 83.0 (C-2′′); δ_H_ 4.03 (H-1′′′′) and δ_C_ 67.8 (C-6′′); δ_H_ 4.21, 4.31 (H_2_-6′′′) and δ_C_ 166.4 (C-9′′′′′) were observed (Figure 2). Consequently, the structure of apetalumoside B_8_ (**2**) was determined as quercetin 3-*O*-[β-d-glucopyranosyl(1→6)]-β-d-(6-*O*-sinapoyl)-glucopyranosyl(1→2)-β-d-glucopyranoside.

**Table 2:** ^1^H and ^13^C-NMR data for **2** in DMSO-*d*_6_ (500 MHz for ^1^H and 125 MHz for ^13^C).

No.	δ_C_	δ_H_ (*J* in Hz)	No.	δ_C_	δ_H_ (*J* in Hz)
2	155.5	-	1′′′	104.3	4.69 (d, 7.5)
3	132.8	-	2′′′	74.3	3.17 (dd, 7.5, 8.5)
4	177.2	-	3′′′	76.2	3.26 (dd, 8.5, 9.0)
5	161.1	-	4′′′	69.4	3.29 (dd, 9.0, 9.0)
6	98.6	6.16 (d, 2.0)	5′′′	73.8	3.51 (m)
7	164.1	-	6′′′	63.0	4.21 (br. d, *ca.* 11)
8	93.4	6.26 (d, 2.0)			4.31 (dd, 5.0, 11.0)
9	156.1	-	1′′′′	103.2	4.03 (d, 7.5)
10	103.7	-	2′′′′	73.2	2.83 (dd, 7.5, 8.5)
1′	121.0	-	3′′′′	76.4	2.96 (dd, 8.5, 8.5)
2′	116.1	7.55 (d, 2.0)	4′′′′	69.6	3.02 (dd, 8.5, 8.5)
3′	144.8	-	5′′′′	76.4	2.87 (m)
4′	148.4	-	6′′′′	60.7	3.40 (dd, 5.0, 11.5)
5′	115.2	6.89 (d, 8.5)			3.55 (br. d, *ca.* 12)
6′	121.8	7.59 (dd, 2.0, 8.5)	1′′′′′	124.2	-
5-OH	-	12.64 (br. s)	2′′′′′,6′′′′′	105.8	6.83 (s)
1′′	98.0	5.65 (d, 7.0)	3′′′′′,5′′′′′	147.8	-
2′′	83.0	3.53 (dd, 7.0, 8.5)	4′′′′′	138.1	-
3′′	76.1	3.52 (dd, 8.5, 9.0)	7′′′′′	145.2	7.39 (d, 16.0)
4′′	69.1	3.27 (dd, 9.0, 9.0)	8′′′′′	114.3	6.30 (d, 16.0)
5′′	76.2	3.32 (m)	9′′′′′	166.4	-
6′′	67.8	3.45 (dd, 5.0, 11.5)	3′′′′′,5′′′′′-OCH_3_	55.9	3.77 (s)
		3.78 (br. d, *ca*. 12)			

*Apetalumoside B_9_* (**3**), ${\left[\text{α}\right]}_{\text{D}}^{25}$ –61.6° (MeOH). Negative-ion HRESI-TOF-MS determination suggested the molecular formula of it was C_55_H_60_O_30_ (*m*/*z* 1199.3095 [M − H]^−^, calcd for C_55_H_59_O_30_ 1199.3097). The proton and carbon signals in ^1^H and ^13^C-NMR spectra (DMSO-*d*_6_, Table 3) were very similar to those of **1**, except for the signals due to the aglycon, quercetin [δ 6.15 (1H, br. s, H-6), 6.20 (1H, br. s, H-8), 6.86 (1H, d, *J* = 8.5 Hz, H-5′), 7.52 (1H, dd, *J* = 1.5, 8.5 Hz, H-6′), 7.57 (1H, d, *J* = 1.5 Hz, H-2′), 12.76 (1H, br. s, 5-OH)]. The linkage positions of sugar parts with aglycon and two sinapoyl groups were elucidated by the HMBC determination (Figure 2), which showed long-range correlations between δ_H_ 5.69 (1H, d, *J* = 7.5 Hz, H-1′′) and δ_C_ 132.6 (C-3); δ_H_ 4.63 (1H, d, *J* = 7.5 Hz, H-1′′′and δ_C_ 82.6 (C-2′′); δ_H_ 4.27 (1H, d, *J* = 8.0 Hz, H-1′′′′) and δ_C_ 66.6 (C-6′′); δ_H_ [4.15 (1H, br. d, *ca. J* = 12 Hz), 4.28 (1H, dd, *J* = 4.5, 11.5 Hz), H_2_-6′′′] and δ_C_ 166.4 (C-9′′′′′); δ_H_ 4.42 (1H, dd, *J* = 8.0, 9.0 Hz, H-2′′′′) and δ_C_ 165.5 (C-9′′′′′′). Meanwhile, the badly overlapped protons in sugar chemical shift range were assigned by HSQC-TOCSY experiment. Finally, the presence of d-glucose was proved by acid analysis [1,2]. Then the structure of apetalumoside B_9_ (**3**) was elucidated as quercetin 3-*O*-[β-d-(2-*O*-sinapoyl)-glucopyranosyl(1→6)]-β-d-(6-*O*-sinapoyl)-glucopyranosyl(1→2)-β-d-glucopyranoside.

**Table 3:** ^1^H and ^13^C-NMR data for **3** in DMSO-*d*_6_ (500 MHz for ^1^H and 125 MHz for ^13^C).

No.	δ_C_	δ_H_ (*J* in Hz)	No.	δ_C_	δ_H_ (*J* in Hz)
2	156.0	-	4′′′	69.4	3.23 (dd, 8.0, 8.0)
3	132.6	-	5′′′	73.8	3.41 (m)
4	177.3	-	6′′′	63.1	4.15 (br. d, *ca.* 12)
5	161.0	-			4.28 (dd, 4.5, 11.5)
6	98.7	6.15 (br. s)	1′′′′	100.1	4.27 (d, 8.0)
7	163.9	-	2′′′′	73.7	4.42 (dd, 8.0, 9.0)
8	93.7	6.20 (br. s)	3′′′′	74.2	2.79 (dd, 9.0, 9.0)
9	156.1	-	4′′′′	69.6	3.15 (dd, 8.0, 9.0)
10	103.9	-	5′′′′	76.3	2.69 (m)
1′	121.0	-	6′′′′	60.1	3.44 (m)
2′	116.1	7.57 (d, 1.5)	1′′′′′	124.2	-
3′	144.7	-	2′′′′′,6′′′′′	105.9	6.79 (s)
4′	148.5	-	3′′′′′,5′′′′′	147.8	-
5′	115.2	6.86 (d, 8.5)	4′′′′′	138.1	-
6′	121.6	7.52 (dd, 1.5, 8.5)	7′′′′′	145.1	7.34 (d, 16.0)
5-OH	-	12.76 (br. s)	8′′′′′	114.3	6.25 (d, 16.0)
1′′	97.9	5.69 (d, 7.5)	9′′′′′	166.4	-
2′′	82.6	3.51 (dd, 7.5, 8.5)	3′′′′′,5′′′′′-OCH_3_	55.9	3.74 (s)
3′′	76.1	3.39 (dd, 8.0, 8.5)	1′′′′′′	124.5	-
4′′	69.2	3.04 (dd, 8.0, 9.0)	2′′′′′′,6′′′′′′	105.9	7.07 (s)
5′′	78.1	3.16 (m)	3′′′′′′,5′′′′′′	148.1	-
6′′	66.6	3.43 (dd, 5.5, 11.5)	4′′′′′′	138.2	-
		3.69 (br. d, *ca.* 12)	7′′′′′′	145.1	7.49 (d, 16.0)
1′′′	104.2	4.63 (d, 7.5)	8′′′′′′	115.0	6.32 (d, 16.0)
2′′′	74.3	3.14 (dd, 7.5, 8.0)	9′′′′′′	165.5	-
3′′′	76.2	3.24 (dd, 8.0, 8.0)	3′′′′′′,5′′′′′′-OCH_3_	56.2	3.88 (s)

*Apetalumoside B_10_* (**4**) was obtained as yellow powder with negative rotation (${\left[\text{α}\right]}_{\text{D}}^{25}$ –66.4°, in MeOH). Acid hydrolysis with 1 M HCl, it gave d-glucose [1,2]. The molecular formula of **4**, C_44_H_50_O_26_ (*m*/*z* 993.2537 [M − H]^−^, calcd for C_44_H_49_O_26_ 993.2518), was the same as that of **2**. And the ^1^H and ^13^C (DMSO-*d*_6_, Table 4) together with various 2D NMR experiments of **4** showed the same fragments {quercetin aglycon [δ 6.39 (1H, d, *J* = 1.5 Hz, H-6), 6.58 (1H, d, *J* = 1.5 Hz, H-8), 6.90 (1H, d, *J* = 8.5 Hz, H-5′), 7.57 (1H, d, *J* = 1.5 Hz, H-2′), 7.61 (1H, dd, *J* = 1.5, 8.5 Hz, H-6′), 12.70 (1H, br. s, 5-OH)], three β-d-glucopyranosyl groups [δ 4.68 (1H, d, *J* = 7.5 Hz, H-1′′′), 5.03 (1H, d, *J* = 7.5 Hz, H-1′′′′), 5.68 (1H, d, *J* = 6.5 Hz, H-1′′)], and a sinapoyl [δ_H_ 3.75 (6H, s, 3′′′′′,5′′′′′-OCH_3_), 6.25 (1H, d, *J* = 15.5 Hz, H-8′′′′′), 6.78 (2H, s, H-2′′′′′,6′′′′′), 7.36 (1H, d, *J* = 15.5 Hz, H-7′′′′′); δ_C_ 166.4 (C-9′′′′′)]} as **2**. But comparison the ^1^H and ^13^C-NMR data of 6–8 positions in **4** [δ_H_ 6.39 (H-6), 6.58 (H-8); δ_C_ 94.0 (C-8), 99.1 (C-6), 162.5 (C-7)] with those in **2** [δ_H_ 6.16 (H-6), 6.26 (H-8); δ_C_ 93.4 (C-8), 98.6 (C-6), 164.1 (C-7)] revealed a glycoside substitution shift around the 7-position. Meanwhile, the ^13^C-NMR data of C-6′′ of **4** (δ_C_ 60.4) shifted to high field compared with that of **2** (δ_C_ 67.8), which meant there was no substitution at C-6′′ position for compound **4**. Furthermore, in the HMBC experiment, long-range correlations were observed between δ_H_ 5.68 (H-1′′) and δ_C_ 133.1 (C-3); δ_H_ 4.68 (H-1′′′) and δ_C_ 83.6 (C-2′′); δ_H_ 5.03 (H-1′′′′) and δ_C_ 162.5 (C-7); δ_H_ [4.20 (1H, br. d, *ca. J* = 12 Hz), 4.31 (1H, dd, *J* = 5.0, 11.5 Hz), H_2_-6′′′] and δ_C_ 166.4 (C-9′′′′′). Consequently, the structure of apetalumoside B_10_ (**4**) was determined as quercetin 3-*O*-β-d-(6-*O*-sinapoyl)-glucopyranosyl(1→2)-β-d-glucopyranoside-7-*O*-β-d-glucopyranoside.

**Table 4:** ^1^H and ^13^C-NMR data for **4** in DMSO-*d*_6_ (500 MHz for ^1^H and 125 MHz for ^13^C).

No.	δ_C_	δ_H_ (*J* in Hz)	No.	δ_C_	δ_H_ (*J* in Hz)
2	156.1	-	1′′′	104.5	4.68 (d, 7.5)
3	133.1	-	2′′′	74.4	3.17 (dd, 7.5, 9.0)
4	177.4	-	3′′′	76.1	3.26 (dd, 8.0, 9.0)
5	160.7	-	4′′′	69.5	3.25 (dd, 8.0, 8.0)
6	99.1	6.39 (d, 1,5)	5′′′	73.8	3.53 (m)
7	162.5	-	6′′′	63.1	4.20 (br. d, *ca.* 12)
8	94.0	6.58 (d, 1.5)			4.31 (dd, 5.0, 11.5)
9	155.7	-	1′′′′	99.6	5.03 (d, 7.5)
10	105.4	-	2′′′′	73.0	3.28 (dd, 7.5, 8.0)
1′	120.8	-	3′′′′	76.2	3.34 (dd, 8.0, 9.0)
2′	116.2	7.57 (d, 1.5)	4′′′′	69.5	3.19 (dd, 9.0, 9.0)
3′	144.8	-	5′′′′	77.0	3.44 (m)
4′	148.7	-	6′′′′	60.5	3.49 (m, overlapped)
5′	115.2	6.90 (d, 8.5)			3.72 (br. d, *ca.* 11)
6′	121.9	7.61 (dd, 1.5, 8.5)	1′′′′′	124.1	-
5-OH	-	12.70 (br. s)	2′′′′′,6′′′′′	105.8	6.78 (s)
1′′	97.7	5.68 (d, 6.5)	3′′′′′,5′′′′′	147.8	-
2′′	83.6	3.49 (m, overlapped)	4′′′′′	138.1	-
3′′	76.3	3.49 (m, overlapped)	7′′′′′	145.1	7.36 (d, 15.5)
4′′	69.4	3.13 (dd, 8.0, 8.0)	8′′′′′	114.3	6.25 (d, 15.5)
5′′	77.4	3.10 (m)	9′′′′′	166.4	-
6′′	60.4	3.27 (m, overlapped)	3′′′′′,5′′′′′-OCH_3_	55.9	3.75 (s)
		3.49 (m, overlapped)			

**Figure 3:**
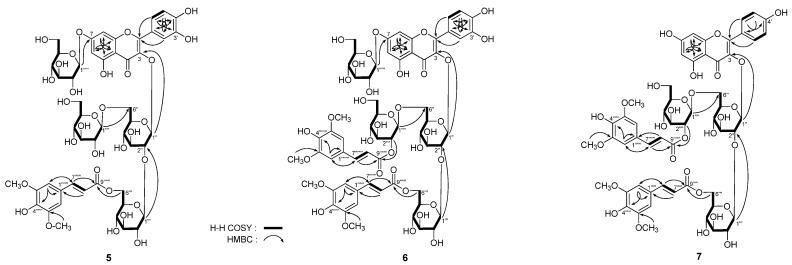
The main ^1^H ^1^H COSY and HMBC correlations of **5**–**7**.

*Apetalumoside B_11_* (**5**) was isolated as yellow powder, too. It had the molecular formula, C_50_H_60_O_31_, deduced from the negative-ion HRESI-TOF-MS (*m*/*z* 1155.3063 [M − H]^−^, calcd for C_50_H_59_O_31_ 1155.3046). On acid hydrolysis and identification with HPLC analysis, the presence of D-glucose was determined [1,2]. The ^1^H and ^13^C (DMSO-*d*_6_, Table 5) together with ^1^H ^1^H COSY, HSQC, HMBC, and HSQC-TOCSY spectra revealed it had the same aglycon, quercetin as **2**–**4** [δ 6.38 (1H, d, *J* = 2.0 Hz, H-6), 6.55 (1H, d, *J* = 2.0 Hz, H-8), 6.88 (1H, d, *J* = 8.5 Hz, H-5′), 7.57 (1H, d, *J* = 2.0 Hz, H-2′), 7.58 (1H, dd, *J* = 2.0, 8.5 Hz, H-6′), 12.63 (1H, br. s, 5-OH)]. On the other hand, there were four β-d-glucopyranosyl groups [δ 3.99 (1H, d, *J* = 7.5 Hz, H-1′′′′), 4.66 (1H, d, *J* = 7.5 Hz, H-1′′′), 5.01 (1H, d, *J* = 7.5 Hz, H-1′′′′′), 5.62 (1H, d, *J* = 6.5 Hz, H-1′′)], and a sinapoyl [δ_H_ 3.75 (6H, s, 3′′′′′′,5′′′′′′-OCH_3_), 6.26 (1H, d, *J* = 16.0 Hz, H-8′′′′′′), 6.80 (2H, s, H-2′′′′′′,6′′′′′′), 7.36 (1H, d, *J* = 16.0 Hz, H-7′′′′′′); δ_C_ 166.4 (C-9′′′′′′)]} in **5**. There was one more β-d-glucopyranosyl in **5** than in **4**. Moreover, the ^13^C-NMR data of C-6′′ for **5** (δ_C_ 67.8) shifted to low field compared with that of **4** (δ_C_ 60.4), which indicated C-6′′ position might be substituted with β-d-glucopyranosyl in **5**. Meanwhile, in the HMBC experiment (Figure 3), long-range correlation was observed between δ_H_ 3.99 (1H, d, *J* = 7.5 Hz, H-1′′′′) and δ_C_ 67.8 (C-6′′). Finally, in the HSQC-TOCSY spectra, the correlations between δ_C_ 97.9 (C-1′′) and δ_H_ 3.25 (H-4′′), 3.32 (H-5′′), 3.50 (H-3′′), 3.52 (H-2′′), 5.62 (H-1′′); δ_H_ 3.78 (H-6b′′) and δ_C_ 67.8 (C-6′′), 69.2 (C-4′′), 76.3 (C-5′′); δ_C_ 104.5 (C-1′′′) and δ_H_ 3.16 (H-2′′′), 3.25 (H-4′′′), 3.26 (H-3′′′), 3.51 (H-5′′′), 4.66 (H-1′′′); δ_H_ 4.19, 4.30 (H_2_-6′′′) and δ_C_ 63.1 (C-6′′′), 69.4 (C-4′′′), 73.8 (C-5′′′); δ_C_ 103.2 (C-1′′′′) and δ_H_ 2.80 (H-2′′′′), 2.85 (H-5′′′′), 2.90 (H-3′′′′), 2.98 (H-4′′′′), 3.99 (H-1′′′′); δ_C_ 60.7 (C-6′′′′) and δ_H_ 2.85 (H-5′′′′), 2.90 (H-3′′′′), 2.98 (H-4′′′′), 3.38, 3.54 (H_2_-6′′′′); δ_C_ 99.8 (C-1′′′′′) and δ_H_ 3.19 (H-4′′′′′), 3.32 (H-3′′′′′), 3.27 (H-2′′′′′), 3.43 (H-5′′′′′), 5.01 (H-1′′′′′); δ_C_ 60.6 (C-6′′′′′) and δ_H_ 3.19 (H-4′′′′′), 3.32 (H-3′′′′′), 3.43 (H-5′′′′′), 3.49, 3.72 (H_2_-6′′′′′) were observed, then the badly overlapped protons in sugar chemical shift range were assigned clearly. On the basis of above mentioned evidence, the structure of **5** was determined to be quercetin 3-*O*-[β-d-glucopyranosyl(1→6)]-β-d-(6-*O*-sinapoyl)-glucopyranosyl(1→2)-β-d-glucopyranoside-7-*O*-β-d-glucopyranoside.

**Table 5:** ^1^H and ^13^C-NMR data for **5** in DMSO-*d*_6_ (500 MHz for ^1^H and 125 MHz for ^13^C).

No.	δ_C_	δ_H_ (*J* in Hz)	No.	δ_C_	δ_H_ (*J* in Hz)
2	156.2	-	4′′′	69.4	3.25 (m, overlapped)
3	133.1	-	5′′′	73.8	3.51 (m)
4	177.3	-	6′′′	63.1	4.19 (br. d, *ca.* 12)
5	160.7	-			4.30 (dd, 4.5, 12.0)
6	99.2	6.38 (d, 2.0)	1′′′′	103.2	3.99 (d, 7.5)
7	162.6	-	2′′′′	73.1	2.80 (dd, 7.5, 8.0)
8	94.3	6.55 (d, 2.0)	3′′′′	76.2	2.90 (dd, 8.0, 9.0)
9	155.7	-	4′′′′	69.6	2.98 (dd, 9.0, 9.0)
10	105.5	-	5′′′′	76.4	2.85 (m)
1′	120.8	-	6′′′′	60.7	3.38 (dd, 5.5, 11.5)
2′	116.3	7.57 (d. 2.0)			3.54 (br. d, *ca.* 12)
3′	144.8	-	1′′′′′	99.8	5.01 (d, 7.5)
4′	148.7	-	2′′′′′	73.1	3.27 (dd, 7.5, 8.0)
5′	115.2	6.88 (d, 8.5)	3′′′′′	76.2	3.32 (dd, 8.0, 9.0)
6′	121.9	7.58 (dd, 2.0, 8.5)	4′′′′′	69.5	3.19 (dd, 9.0, 9.0)
5-OH	-	12.63 (br. s)	5′′′′′	77.0	3.43 (m)
1′′	97.9	5.62 (d, 6.5)	6′′′′′	60.6	3.49 (dd, 4.0, 11.5)
2′′	83.2	3.52 (dd, 6.5, 8.0)			3.72 (br. d, *ca.* 12)
3′′	76.1	3.50 (dd, 8.0, 8.0)	1′′′′′′	124.2	-
4′′	69.2	3.25 (m, overlapped)	2′′′′′′,6′′′′′′	105.9	6.80 (s)
5′′	76.3	3.32 (m)	3′′′′′′,5′′′′′′	147.8	-
6′′	67.8	3.43 (dd, 5.5, 11.5)	4′′′′′′	138.1	-
		3.78 (dd, 3.5, 11.5)	7′′′′′′	145.1	7.36 (d, 16.0)
1′′′	104.5	4.66 (d, 7.5)	8′′′′′′	114.3	6.26 (d, 16.0)
2′′′	74.4	3.16 (dd, 7.5, 8.0)	9′′′′′′	166.4	-
3′′′	76.2	3.26 (m, overlapped)	3′′′′′′,5′′′′′′-OCH_3_	55.9	3.75 (s)

*Apetalumoside B_12_* (**6**), ${\left[\text{α}\right]}_{\text{D}}^{25}$ –84.0° (MeOH), was isolated as yellow powder. The molecular formula, C_61_H_70_O_35_, of **6** was determined from negative-ion HRESI-TOF-MS (*m*/*z* 1361.3625 [M − H]^−^, calcd for C_61_H_69_O_35_ 1361.3625). Acid hydrolysis of **6** with 1 M HCl liberated D-glucose [1,2]. Comparison the ^1^H and ^13^C (DMSO-*d*_6_, Table 6) spectra with those of **5**, revealed there was another sinapoyl [δ_H_ 3.87 (6H, s, 3′′′′′′′,5′′′′′′′-OCH_3_), 6.32 (1H, d, *J* = 16.0 Hz, H-8′′′′′′′), 7.06 (2H, s, H-2′′′′′′′,6′′′′′′′), 7.50 (1H, d, *J* = 16.0 Hz, H-7′′′′′′′); δ_C_ 165.5 (C-9′′′′′′′)] appeared in **6**, and the ^1^H-NMR data of 2′′′′-position [δ_H_ 4.39 (1H, dd, *J* = 8.0, 8.0 Hz, H-2′′′′)] shifted to low field relatived to that of **5** [δ_H_ 2.80 (1H, dd, *J* = 7.5, 8.0 Hz, H-2′′′′). The above mentioned evidence suggested the another sinapoyl group linked with 2′′′′-position, which was certified by the long-range correlation between δ_H_ 4.39 (H-2′′′′) and δ_C_ 165.5 (C-9′′′′′′′) observed in the HMBC experiment. In conjunction with analysis of HSQC and HSQC-TOCSY spectra, the ^1^H and ^13^C-NMR data for **6** were assigned. Finally, the structure of apetalumoside B_12_ (**6**) was clarified to be quercetin 3-*O*-[β-d-(2-*O*-sinapoyl)-glucopyranosyl(1→6)]-β-d-(6-*O*-sinapoyl)-glucopyranosyl(1→2)-β-d-glucopyranoside-7-*O*-β-d-glucopyranoside.

**Table 6:** ^1^H and ^13^C-NMR data for **6** in DMSO-*d*_6_ (500 MHz for ^1^H and 125 MHz for ^13^C).

No.	δ_C_	δ_H_ (*J* in Hz)	No.	δ_C_	δ_H_ (*J* in Hz)
2	156.8	-	1′′′′	100.2	4.22 (d, 8.0)
3	132.8	-	2′′′′	73.4	4.39 (dd, 8.0, 8.0)
4	177.4	-	3′′′′	74.0	2.71 (dd, 8.0, 8.0)
5	160.8	-	4′′′′	69.6	3.11 (dd, 8.0, 9.5)
6	99.3	6.40 (d, 1.5)	5′′′′	76.3	2.68 (m)
7	162.7	-	6′′′′	60.1	3.44 (m, overlapped)
8	94.8	6.51 (d, 1.5)			3.48 (m, overlapped)
9	155.5	-	1′′′′′	100.0	5.00 (d, 7.5)
10	105.5	-	2′′′′′	73.1	3.26 (dd, 7.5, 7.5)
1′	120.7	-	3′′′′′	76.2	3.30 (dd, 7.5, 8.5)
2′	116.3	7.58 (d, 1.5)	4′′′′′	69.6	3.16 (dd, 7.5, 8.5)
3′	144.8	-	5′′′′′	77.0	3.40 (m)
4′	148.8	-	6′′′′′	60.6	3.48 (m, overlapped)
5′	115.2	6.86 (d, 8.5)			3.75 (br. d, *ca.* 12)
6′	121.7	7.51 (dd, 1.5, 8.5)	1′′′′′′	124.1	-
5-OH	-	12.76 (br. s)	2′′′′′′,6′′′′′′	105.8	6.76 (s)
1′′	97.8	5.67 (d, 7.5)	3′′′′′′,5′′′′′′	147.8	-
2′′	82.8	3.48 (m, overlapped)	4′′′′′′	138.1	-
3′′	76.0	3.38 (dd, 7.5, 8.0)	7′′′′′′	145.2	7.33 (d, 16.0)
4′′	69.1	3.01 (dd, 8.0, 9.0)	8′′′′′′	114.3	6.22 (d, 16.0)
5′′	78.1	3.14 (m)	9′′′′′′	166.3	-
6′′	66.7	3.45 (m, overlapped)	3′′′′′′,5′′′′′′-OCH_3_	55.9	3.73 (s)
		3.67 (br. d, *ca.* 13)	1′′′′′′′	124.5	-
1′′′	104.4	4.61 (d, 7.5)	2′′′′′′′,6′′′′′′′	105.9	7.06 (s)
2′′′	74.4	3.13 (dd, 7.5, 8.0)	3′′′′′′′,5′′′′′′′	148.1	-
3′′′	76.2	3.24 (dd, 8.0, 8.0)	4′′′′′′′	138.2	-
4′′′	69.5	3.23 (dd, 8.0, 8.0)	7′′′′′′′	145.1	7.50 (d, 16.0)
5′′′	73.8	3.41 (m)	8′′′′′′′	114.9	6.32 (d, 16.0)
6′′′	63.2	4.15 (br. d, *ca.* 11)	9′′′′′′′	165.5	-
		4.28 (dd, 6.5, 11.0)	3′′′′′′′,5′′′′′′′-OCH_3_	56.1	3.87 (s)

*Apetalumoside C_1_* (**7**), ${\left[\text{α}\right]}_{\text{D}}^{25}$ –47.2° (MeOH). Negative-ion HRESI-TOF-MS determination suggested the molecular formula of it was C_55_H_60_O_29_ (*m*/*z* 1183.3123 [M − H]^−^, calcd for C_55_H_59_O_29_ 1183.3147). Treated **7** with 1 M HCl to yield D-glucose [1,2]. The proton and carbon signals in ^1^H and ^13^C-NMR spectra (DMSO-*d*_6_, Table 7) were very similar to those of **1**, except for the signals due to the aglycon, kaempferol [δ 6.14 (1H, br. s, H-6), 6.27 (1H, br. s, H-8), 6.85 (2H, d, *J* = 8.5 Hz, H-3′,5′), 7.93 (2H, d, *J* = 8.5 Hz, H-2′,6′), 12.72 (1H, br. s, 5-OH)]. The linkage positions of sugar parts with aglycon and two sinapoyl groups were determined by the HMBC experiment, which showed long-range correlations between δ_H_ 5.58 (1H, d, *J* = 6.5 Hz, H-1′′) and δ_C_ 132.5 (C-3); δ_H_ 4.62 (1H, d, *J* = 7.5 Hz, H-1′′′) and δ_C_ 82.0 (C-2′′); δ_H_ 4.26 (1H, d, *J* = 8.0 Hz, H-1′′′′) and δ_C_ 66.7 (C-6′′); δ_H_ [4.17 (1H, br. d, *ca. J* = 12 Hz), 4.28 (1H, dd, *J* = 5.5, 11.5 Hz), H_2_-6′′′] and δ_C_ 166.4 (C-9′′′′′); δ_H_ 4.43 (1H, dd, *J* = 8.0, 9.0 Hz, H-2′′′′) and δ_C_ 165.4 (C-9′′′′′′). Moreover, the ^1^H-NMR data for four β-d-glucopyranosyl groups were assigned by HSQC and HSQC-TOCSY determination. On the basis of above mentioned evidence, the structure of apetalumoside C_1_ (**7**) was elucidated as kaempferol 3-*O*-[β-d-(2-*O*-sinapoyl)-glucopyranosyl(1→6)]-β-d-(6-*O*-sinapoyl)-glucopyranosyl(1→2)-β-d-glucopyranoside.

**Table 7:** ^1^H and ^13^C-NMR data for **7** in DMSO-*d*_6_ (500 MHz for ^1^H and 125 MHz for ^13^C).

No.	δ_C_	δ_H_ (*J* in Hz)	No.	δ_C_	δ_H_ (*J* in Hz)
2	156.1	-	6′′′	63.2	4.17 (br. d, *ca*. 12)
3	132.5	-			4.28 (dd, 5.5, 11.5)
4	177.3	-	1′′′′	100.2	4.26 (d, 8.0)
5	161.1	-	2′′′′	73.6	4.43 (dd, 8.0, 9.0)
6	98.9	6.14 (br. s)	3′′′′	74.2	2.86 (dd, 9.0, 9.0)
7	164.5	-	4′′′′	69.9	3.11 (dd, 9.0, 9.0)
8	93.8	6.27 (br. s)	5′′′′	76.5	2.78 (m)
9	156.2	-	6′′′′	60.4	3.40 (m, overlapped)
10	103.7	-			3.52 (br. d, *ca.* 12)
1′	120.6	-	1′′′′′	124.2	-
2′,6′	130.7	7.93 (d, 8.5)	2′′′′′,6′′′′′	106.0	6.79 (s)
3′,5′	115.1	6.85 (d, 8.5)	3′′′′′,5′′′′′	147.8	-
4′	159.9	-	4′′′′′	138.2	-
5-OH	-	12.72 (br. s)	7′′′′′	145.1	7.37 (d, 15.5)
1′′	97.9	5.58 (d, 6.5)	8′′′′′	114.4	6.27 (d, 15.5)
2′′	82.0	3.41 (dd, 6.5, 8.0)	9′′′′′	166.4	-
3′′	76.2	3.40 (dd, 8.0, 9.0)	3′′′′′,5′′′′′-OCH_3_	55.9	3.73 (s)
4′′	69.2	3.01 (dd, 9.0, 9.0)	1′′′′′′	124.5	-
5′′	77.8	3.13 (m)	2′′′′′′,6′′′′′′	106.0	7.05 (s)
6′′	66.7	3.44 (dd, 6.5, 12.0)	3′′′′′′,5′′′′′′	148.1	-
		3.68 (br. d, *ca.* 12)	4′′′′′′	138.3	-
1′′′	103.9	4.62 (d, 7.5)	7′′′′′′	145.1	7.49 (d, 16.0)
2′′′	74.3	3.11 (dd, 7.5, 9.0)	8′′′′′′	115.0	6.29 (d, 16.0)
3′′′	76.3	3.21 (dd, 9.0, 9.0)	9′′′′′′	165.4	-
4′′′	69.5	3.23 (dd, 9.0, 9.0)	3′′′′′′,5′′′′′′-OCH_3_	56.1	3.87 (s)
5′′′	73.8	3.35 (m)			

## 3. Experimental

### 3.1. General

UV spectra were recorded on a Varian Cary 50 UV-Vis spectrophotometer (Varian, Inc., Hubbardsdon, MA, USA). IR spectra were obtained on a Varian 640-IR FT-IR spectrophotometer (Varian Australia Pty Ltd, Mulgrave, Australia). Optical rotations were determined on a Rudolph Autopol^®^ IV automatic polarimeter (Rudolph Research Analytical, Hackettstown NJ, USA). NMR spectra were measured on a Bruker AVANCE III 500 MHz NMR spectrometer (500 MHz for ^1^H and 125 MHz for ^13^C-NMR, Bruker BioSpin AG Industriestrasse 26 CH-8117, Fällanden, Switzerland) with TMS as an internal standard. Negative-ion HRESI-TOF-MS were determined on an Agilent 6520 Accurate-Mass Q-Tof MS spectrometer (drying gas, N_2_; flow rate, 8.0 L/min; temperature, 350 °C; nebulizer, 30 psig; capillary, −3500 V; fragmentor, 175 V; skimmer, 65 V; OCT RF V, 750 V. Mass range recorded *m*/*z* 100–1200, Agilent Technologies, Inc., Santa Clara, CA, USA).

Column chromatographies were performed on macroporous resin D101 (Haiguang Chemical Co., Ltd., Tianjin, China), Silica gel (48–75 μm, Qingdao Haiyang Chemical Co., Ltd., Qingdao, China), ODS (40–63 μm, YMC Co., Ltd., Tokyo, Japan), and Sephadex LH-20 (Ge Healthcare Bio-Sciences, Uppsala, Sweden), and Preparative HPLC (PHPLC) column, Cosmosil 5C18-MS-II (20 mm i.d. × 250 mm, 5 μM, Nakalai Tesque, Inc., Tokyo, Japan) were used to purify the constituents.

### 3.2. Plant Material

The seeds of *L. apetalum* were collected from Anguo city, China, and identified by Li Tianxiang. The voucher specimen was deposited at the Academy of Traditional Chinese Medicine of Tianjin University of TCM (No. 20120501).

### 3.3. Extraction and Isolation

*L. apetalum* seeds (10 kg) were crushed and refluxed with 50% ethanol/water. Then, the 50% ethanol/water extract was partitioned in a CHCl_3_/H_2_O mixture (1:1, *v*/*v*), and CHCl_3_ and H_2_O layers were obtained. Then the H_2_O layer was subjected to D101 macroporous resin CC (H_2_O → 95% EtOH). As a result, H_2_O and 95% EtOH eluted fractions were given.

The EtOH fraction (80 g) was subjected to silica gel CC [CHCl_3_ → CHCl_3_/MeOH (100:3 → 100:5, *v*/*v*) → CHCl_3_/MeOH/H_2_O (10:3:1 → 6:4:1, *v*/*v*/*v*) → MeOH] to yield 16 fractions (Fr. 1–16). Fraction 12 was isolated by ODS CC [MeOH/H_2_O (10:90 → 20:80 → 30:70 → 40:60 → 50:50 → 70:30→ 100:0, *v*/*v*] to give 9 fractions (Fr. 12-1–12-9). Fraction 12-6 was separated by PHPLC [CH_3_CN/1% CH_3_COOH (18:82 → 100:0, *v*/*v*)] to obtain 14 fractions (Fr. 12-6-1–12-6-14). Fraction 12-6-11 was purified by PHPLC [CH_3_CN/1% CH_3_COOH (20:80, *v*/*v*)], and apetalumoside B_9_ (**3**, 16.1 mg) was obtained. Fraction 12-6-12 was isolated by PHPLC [CH_3_CN/1% CH_3_COOH (18:82, *v*/*v*)] to yield apetalumosides A_1_ (**1**, 23.7 mg) and C_1_ (**7**, 15.6 mg) respectively. Fraction 14 was separated by Sephadex LH-20 CC [MeOH/H_2_O (1:1, *v*/*v*)] to yield 7 fractions (Fr. 14-1–14-7). Fraction 14-5 was purified by PHPLC [CH_3_CN/1% CH_3_COOH (15:85, *v*/*v*)], as a result, 18 fractions (Fr. 14-5-1–14-5-18) were obtained. Fraction 14-5-5 was subjected to PHPLC [CH_3_CN/1% CH_3_COOH (14:86, *v*/*v*)] to give apetalumoside B_12_ (**6**, 11.4 mg). Fraction 14-5-7 was separated by Sephadex LH-20 CC [MeOH/H_2_O (1:1, *v*/*v*)] to afford three fractions (Fr. 14-5-7-1–14-5-7-3). Fraction 14-5-7-2 was subjected to PHPLC [CH_3_CN/1% CH_3_COOH (12:88, *v*/*v*)], and apetalumoside B_10_ (**4**, 19.8 mg) was obtained. Fraction 14-5-15 was further separated by PHPLC [MeOH/1% CH_3_COOH (34:66, *v*/*v*)] to give apetalumoside B_8_ (**2**, 50.2 mg). Fraction 16 was separated by PHPLC through gradient elution [MeOH/1% CH_3_COOH (20:80 → 25:75 → 30:70 → 35:65 → 40:60 → 50:50 → 60:40 → 100:0, *v*/*v*)] to obtain 26 fractions (Fr. 16-1–16-26). Fraction 16-10 was further isolated by PHPLC [CH_3_CN/1% CH_3_COOH (12:88, *v*/*v*)] to yield three fractions (Fr. 16-10-1–16-10-3). Fraction 16-10-2 was purified by Sephadex LH-20 CC [MeOH/H_2_O (1:1, *v*/*v*)], and apetalumoside B_11_ (**5**, 16.8 mg) was given.

*Apetalumoside A_1_* (**1**): Yellow powder. ${\left[\text{α}\right]}_{\text{D}}^{25}$ –49.0° (*c* = 0.97, MeOH); IR ν_max_ (KBr) cm^−1^: 3394, 2933, 1701, 1648, 1630, 1604, 1514, 1458, 1356, 1284, 1175, 1114, 1075, 826; UV λ_max_ (MeOH) nm (log ε): 329 (4.56), 266 (4.26, sh), 236 (4.56). ^1^H-NMR (500 MHz, DMSO-*d*_6_) and ^13^C-NMR (125 MHz, DMSO-*d*_6_) spectroscopic data, see Table 1. HRESI-TOF-MS: Negative-ion mode *m*/*z* 1213.3238 [M − H]^−^ (calcd for C_56_H_61_O_30_ 1213.3253).

*Apetalumoside B_8_* (**2**): Yellow powder. ${\left[\text{α}\right]}_{\text{D}}^{25}$ –41.6° (*c* = 0.99, MeOH); IR ν_max_ (KBr) cm^−1^: 3367, 2936, 1700, 1650, 1628, 1606, 1514, 1456, 1361, 1287, 1196, 1077, 823, 597, 521; UV λ_max_ (MeOH) nm (log ε): 334 (4.40), 266 (4.22, sh), 241 (4.38). ^1^H-NMR (500 MHz, DMSO-*d*_6_) and ^13^C-NMR (125 MHz, DMSO-*d*_6_) spectroscopic data, see Table 2. HRESI-TOF-MS: Negative-ion mode *m*/*z* 993.2522 [M − H]^−^ (calcd for C_44_H_49_O_26_ 993.2518).

*Apetalumoside B_9_* (**3**): Yellow powder. ${\left[\text{α}\right]}_{\text{D}}^{25}$ –61.6° (*c* = 0.98, MeOH); IR ν_max_ (KBr) cm^−1^: 3394, 2942, 1700, 1650, 1631, 1605, 1514, 1457, 1362, 1285, 1173, 1115, 1077, 827; UV λ_max_ (MeOH) nm (log ε): 329 (4.60), 269 (4.26, sh), 238 (4.57). ^1^H-NMR (500 MHz, DMSO-*d*_6_) and ^13^C-NMR (125 MHz, DMSO-*d*_6_) spectroscopic data, see Table 3. HRESI-TOF-MS: Negative-ion mode *m*/*z* 1199.3095 [M − H]^−^ (calcd for C_55_H_59_O_30_ 1199.3097).

*Apetalumoside B_10_* (**4**): Yellow powder. ${\left[\text{α}\right]}_{\text{D}}^{25}$ –66.4° (*c* = 0.98, MeOH); IR ν_max_ (KBr) cm^−1^: 3366, 2925, 1698, 1652, 1628, 1601, 1515, 1456, 1342, 1283, 1200, 1075, 824; UV λ_max_ (MeOH) nm (log ε): 333 (4.34), 269 (4.20, sh), 242 (4.36). ^1^H-NMR (500 MHz, DMSO-*d*_6_) and ^13^C-NMR (125 MHz, DMSO-*d*_6_) spectroscopic data, see Table 4. HRESI-TOF-MS: Negative-ion mode *m*/*z* 993.2537 [M − H]^−^ (calcd for C_44_H_49_O_26_ 993.2518).

*Apetalumoside B_11_* (**5**): Yellow powder. ${\left[\text{α}\right]}_{\text{D}}^{25}$ –47.6° (*c* = 0.10, MeOH); IR ν_max_ (KBr) cm^−1^: 3367, 2923, 1702, 1652, 1633, 1601, 1515, 1456, 1343, 1284, 1199, 1075, 824; UV λ_max_ (MeOH) nm (log ε): 333 (4.44), 268 (4.32, sh), 241 (4.45). ^1^H-NMR (500 MHz, DMSO-*d*_6_) and ^13^C-NMR (125 MHz, DMSO-*d*_6_) spectroscopic data, see Table 5. HRESI-TOF-MS: Negative-ion mode *m*/*z* 1155.3063 [M − H]^−^ (calcd for C_50_H_59_O_31_ 1155.3046).

*Apetalumoside B_12_* (**6**): Yellow powder. ${\left[\text{α}\right]}_{\text{D}}^{25}$ –84.0° (*c* = 0.99, MeOH); IR ν_max_ (KBr) cm^−1^: 3367, 2924, 1700, 1654, 1631, 1600, 1515, 1457, 1342, 1281, 1176, 1073, 825; UV λ_max_ (MeOH) nm (log ε): 330 (4.52), 270 (4.24, sh), 238 (4.50). ^1^H-NMR (500 MHz, DMSO-*d*_6_) and ^13^C-NMR (125 MHz, DMSO-*d*_6_) spectroscopic data, see Table 6. HRESI-TOF-MS: Negative-ion mode *m*/*z* 1361.3625 [M − H]^−^ (calcd for C_61_H_69_O_35_ 1361.3625).

*Apetalumoside C_1_* (**7**): Yellow powder. ${\left[\text{α}\right]}_{\text{D}}^{25}$ –47.2° (*c* = 0.77, MeOH); IR ν_max_ (KBr) cm^−1^: 3391, 2936, 1700, 1654, 1628, 1606, 1514, 1457, 1360, 1283, 1178, 1114, 1076, 831; UV λ_max_ (MeOH) nm (log ε): 325 (4.47), 265 (4.20), 238 (4.44, sh). ^1^H-NMR (500 MHz, DMSO-*d*_6_) and ^13^C-NMR (125 MHz, DMSO-*d*_6_) spectroscopic data, see Table 7. HRESI-TOF-MS: Negative-ion mode *m*/*z* 1183.3123 [M − H]^−^ (calcd for C_55_H_59_O_29_ 1183.3147).

Acid Hydrolysis of **1**–**7**: A solution of **1**–**7** (each 1.5 mg) in 1 M HCl (1 mL) was heated under reflux for 3 h, respectively. The reaction mixture was dealt, then analyzed by CH_3_CN/H_2_O (70:30, *v*/*v*; flow rate 1.0 mL/min) using the same condition as reference [1]. As result, d-glusose was detected from **1**–**7** by comparison of its retention time and optical rotation with that of authentic sample (*t*_R_ 8.8 min, positive).

## 4. Conclusions

As results, seven new sinapoyl acylated flavonoid glycosides were obtained from *L. apetalum* seeds. Although various acylated flavonol glycosides distribute widely in the plant kingdom, sinapoylates such as the flavonoid glycosides reported in this paper are quite rare, which were found only in 21 species from eight family plants, including Cruciferae [3,4,5,6,7,8,9,10,11,12,13], Leguminosae [14,15], Apocynaceae [16], Solanaceae [17], Elaeagnaceae [18,19], Rubiaceae [20,21], Ranunculaceae [22], and Moraceae [23] until now. And almost of them were obtained from the Cruciferae family, which includes *L. apetalum* researched by our lab. This will have some guidance for plant taxonomy.
